# Mindfulness in Mental Health and Psychiatric Disorders of Children and Adolescents: A Systematic Review and Meta-Analysis of Randomized Controlled Trials

**DOI:** 10.3390/pediatric17030059

**Published:** 2025-05-14

**Authors:** Bruno Daniel Carneiro, Daniel Humberto Pozza, José Tiago Costa-Pereira, Isaura Tavares

**Affiliations:** 1 Department of Biomedicine, Unit of Experimental Biology, Faculty of Medicine, University of Porto, 4200-319 Porto, Portugal; bcarneiro@med.up.pt (B.D.C.); dhpozza@gmail.com (D.H.P.); jcostapereira@fcna.up.pt (J.T.C.-P.); 2 Rheumatology Service, Unidade Local de Saúde do Alto Minho, Hospital Conde de Bertiandos, 4990-078 Ponte de Lima, Portugal; 3 Institute for Research and Innovation in Health and IBMC (i3S), University of Porto, 4200-135 Porto, Portugal; 4 Faculty of Nutrition and Food Sciences, University of Porto, 4150-180 Porto, Portugal

**Keywords:** mindfulness, mental health, psychiatric disorders, children, adolescents, randomized controlled trials, mindfulness-based stress reduction, mindfulness-based cognitive therapy, anxiety, depression

## Abstract

Mindfulness-Based Interventions (MBIs) are important tools to address mental health issues in children and adolescents. However, previous studies provided variable results that suggest that the effectiveness of those third-wave Cognitive Behavioral Therapies remains uncertain. Objectives: The main objective is to assess the impact of MBIs on anxiety, depression, and stress in children and adolescents. The secondary objective is to examine the modalities of MBIs used, the duration of interventions, and potential confounding factors, such as age. Methods: A comprehensive search of multiple databases was conducted to identify randomized clinical trials (RCTs) evaluating the effects of MBIs on mental health outcomes in children and adolescents. The research was registered in PROSPERO, adhered to PRISMA guidelines, employed the Cochrane Risk of Bias 2 tool, and calculated the effect sizes using mean differences. Results: Thirteen RCTs were included; ten were identified as having some concerns, while three were classified as having a low risk of bias. Mindfulness-Based Stress Reduction (MBSR) demonstrated a small positive effect on depression and anxiety, while non-specific MBIs showed a moderate positive effect both on depression and anxiety. Mindfulness-Based Cognitive Therapy (MBCT) was effective in reducing anxiety, depression (moderate positive effects on both), and stress symptoms. In one study, no significant improvements were seen on both anxiety and depression (for MBCT) and in another study on anxiety (for MBCT/MBSR). The meta-analysis did not identify a significant effect of mindfulness interventions on depression or anxiety. The high heterogeneity suggests varying outcomes, requiring further study of moderating factors. Conclusions: While some studies suggest benefits from MBIs, mainly MBCT’s ability to improve mental health outcomes in children and adolescents, their overall efficacy remains uncertain due to the high heterogeneity. The findings underscore the importance of considering the intervention type, duration, and moderating factors, such as age, when implementing MBIs.

## 1. Introduction

Mindfulness has been defined as the awareness that arises through paying attention in a particular way: on purpose, in the present moment, and non-judgmentally [1]. Mindfulness has gained considerable attention in both clinical and educational contexts. Originally derived from ancient Eastern philosophies and Buddhist meditation practices [2,3], mindfulness has evolved into a therapeutic tool with increasing applications in contemporary medicine. Jon Kabat-Zinn played a pivotal role in bringing mindfulness into clinical and educational settings through his development of the Mindfulness-Based Stress Reduction (MBSR) program in the late 1970s. His work demonstrated mindfulness as a secular approach to reducing stress and improving well-being, inspiring widespread adoption of Mindfulness-Based Interventions (MBIs) for addressing psychological challenges like anxiety, depression, and emotional dysregulation [4].

In recent years, MBIs have been increasingly recognized for their potential to improve mental health, emotional well-being, and cognitive functioning, particularly in children and adolescents, who are at a heightened risk for developing psychiatric disorders. This is especially relevant given that anxiety, depression, and stress in youth have become widespread, with approximately 25% to 31% of adolescents worldwide experiencing these conditions [5,6]. Furthermore, it is known that affective temperaments influence psychological vulnerability and treatment responsiveness in youth [7]. The rise in mental health challenges during childhood and adolescence underscores the need for accessible, evidence-based interventions that can be implemented early to prevent long-term psychological issues and improve overall well-being [5]. The most widely used mindfulness programs include MBSR and Mindfulness-Based Cognitive Therapy (MBCT). MBSR supports individuals in managing a range of challenges by promoting body awareness and acceptance [8,9,10,11,12,13]. MBCT integrates cognitive therapy and psychoeducation to encourage the acceptance of distressing thoughts and emotions, with a focus on metacognitive awareness [9,13], which may help prevent the recurrence of depression [14,15]. MBSR is the prototypical mindfulness intervention originally conceived for treatment of stress and anxiety while MBCT was modelled after MBSR and combines Cognitive Behavioral Therapy (CBT) with mindfulness [16]. Subsequently to the introduction of the MBSR and MBCT programs, other therapies were also designed, namely Mindfulness Self-Compassion (MSC) and, more recently, Mindfulness-Based Relapse Prevention (MBRP) and Mindfulness-Oriented Recovery Enhancement (MORE), with the latter two being for specific clinical contexts. MSC equips individuals with strategies for proactive self-care, facilitating relief from suffering, including chronic pain [15,17,18]. MBRP aims to reduce the likelihood and severity of a relapse by identifying risk factors, while enhancing awareness, exposure, and behavioral flexibility in daily emotional and cognitive processes [19,20]. MORE applies social–behavioral learning theory to enhance participant motivation and engagement. By combining elements of CBT, psychological concepts, and mindfulness, this program addresses psychiatric symptoms, physical pain, and addictive behaviors in a group setting [21,22,23,24].

However, the impact of Mindfulness-Based Interventions (MBIs) on children and adolescents remains debated due to the heterogeneity of the research, including variations in intervention designs, participant characteristics, outcome measures, and program types [25,26]. While some meta-analyses have reported significant positive effects of mindfulness on anxiety, depression, and stress resilience [27,28], others have raised questions about the true efficacy of mindfulness when compared to other widely used interventions, such as Cognitive Behavioral Therapy [29,30]. Additionally, research has indicated that the benefits of mindfulness may not be sustained in the long term, or that the effects are relatively modest in comparison to other therapeutic approaches [25,30].

This variability in findings calls for a more comprehensive and rigorous synthesis of the existing literature to better understand the potential and limitations of mindfulness for addressing mental health issues in youth. In this context, this systematic review and meta-analysis aimed to update and evaluate the effectiveness of MBIs in promoting mental health among children and adolescents, focusing on anxiety, depression, and stress. It also examined the types of MBIs, intervention durations, and potential moderating factors like age.

## 2. Materials and Methods

This systematic review adhered to the Preferred Reporting Items for Systematic Reviews and Meta-Analyses (PRISMA) 2020 guidelines [31]. We followed the PROSPERO database protocol model to ensure study transparency and reproducibility (ID: CRD42024608321). The protocol can be consulted on the PROSPETO database and has been reviewed and approved by all the study authors. The review PICO question was “In children and adolescents with psychiatric disorders or mental health symptoms (P), how effective are Mindfulness-Based Interventions (I), compared to usual care or other psychological interventions (C), in improving mental health outcomes and reducing symptoms of psychiatric disorders (O)?”.

A search in five electronic bibliographic databases, which included Web of Science, PubMed, Scopus, CENTRAL (Cochrane Central Register of Controlled Trials), and PsycInfo was carried out in October 2024. The search strategy was built up combining the following terms: “Mindfulness”[Mesh] AND (“Mental Health”[Mesh] OR “Mental Disorders”[Mesh]) AND (“Adolescent”[Mesh] OR “Child”[Mesh]).

The inclusion criteria were as follows: (1) population include children and adolescents aged < 18 years (not inclusive) with clinical diagnosis of psychiatric disorders (namely anxiety disorder, depression disorder, and stress) or reported symptoms of mental health problems (namely anxiety, depression and stress); (2) defined intervention, namely MBSR, MBCT, MBRP, and MORE, against a defined comparator (usual care, waitlist control, placebo, or other psychological interventions, namely CBT not involving mindfulness interventions); (3) well-defined primary outcomes and secondary outcomes; (4) published RCTs in peer-reviewed journals with more than 10 patients; and (5) use of standardized outcome measures related to mental health, namely measures of stress, anxiety, and depression. The exclusion criteria were as follows: (1) intervention not focused on MBIs; (2) mental health disorders other than anxiety, depression, or stress; and (3) subjects diagnosed with COVID-19 at the time of this study.

Screening of titles and abstracts of identified records was carried out independently by two reviewers to determine eligibility in the Rayyan tool [32]. The “blind mode” was on to ensure that reviewers were not influenced by each other’s decisions, thereby maintaining objectivity and minimizing bias in the screening process. Full texts of potentially relevant studies were then assessed for a comprehensive analysis. A third member of the research team helped to resolve any conflicts. The level of agreement between the authors was assessed using the Kappa test [33].

Data from eligible studies were extracted. The information collected from each study included data about authors, year, country, number of participants, age of participants, intervention, control, period of intervention, outcome measure, effect size, and effectiveness of mindfulness interventions. The risk of bias of the clinical trials was evaluated with the Cochrane RoB 2 tool [34] at the outcome level visualized with the Cochrane risk of bias VISualization app 4.0 [35].

A narrative synthesis with meta-analysis was conducted to summarize the findings of the included studies. The meta-analysis was conducted to assess the effect of mindfulness interventions on depression and anxiety, by using RStudio software (version 2024.12.0+467). A random-effects model was applied as it provides a more conservative estimate effect. The confidence intervals (CIs) were set at 95%. We assessed heterogeneity using τ^2^ and I^2^ statistics. Subgroup meta-analysis was used to analyze the specific effects of MBCT and MBSR interventions on depression and anxiety. Initially 11 studies were included on overall analysis; however, subgroup analyses were restricted to 10 studies that explicitly employed MBCT or MBSR interventions. Meta-analytic results were presented as forest plots displaying effect estimates and corresponding 95% CIs for individual studies.

All the aforementioned statistical techniques that led to the results of this systematic review and meta-analysis were carried out by a team of authors with recognized experience in medical education and in carrying out other systematic reviews and meta-analyses, most of whom have a PhD, and are therefore experienced in working with the aforementioned tools.

## 3. Results

**Figure 1:**
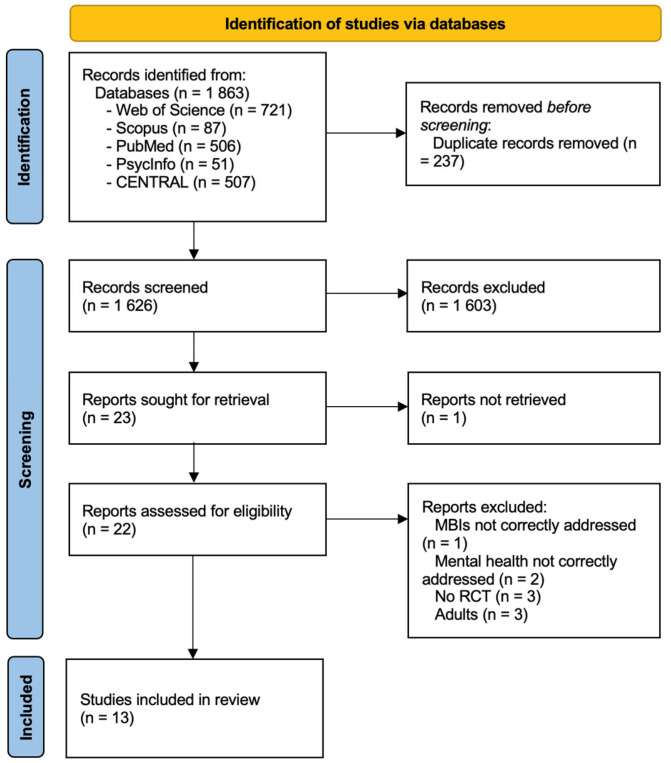
PRISMA flow diagram outlining selection of the included studies.

A comprehensive literature search in the five databases resulted in 1863 potential records identified. Following the removal of duplicate records, 1626 manuscripts remained for the title and abstract review. Of the articles selected, a total of 1603 records were excluded after screening by title and abstract. A total of 22 articles were selected for full-text examination, because 1 article [36] was not retrieved (we tried to contact the author several times without success). The final inclusion criteria were met by 13 records. A concordance index of 95% (Kappa coefficient of 0.95) were obtained between the authors, indicating an almost perfect agreement between the reviewers. Figure 1 shows the entire search process, including the reasons for manuscript exclusion during the full text examination process. The summary of findings is shown in Table 1.

### 3.1. Description of Included Studies

The extracted data characteristics for each study are available in Table 1. The 13 studies included in this review assessed the effectiveness of MBSR (*n* = 4), MBCT (*n* = 7), MBSR and MBCT (*n* = 1), and MBIs not specified (*n* = 1). The following main results extracted from RCTs were divided according to the intervention under study.

#### 3.1.1. MBSR

Folch et al. conducted a study in Spain with students aged 9 to 11, showing a medium positive effect in reducing anxiety, as evaluated using the neuropsychological assessment questionnaire SPECI (Screening for Children’s Emotional and Behavioral Problems) [37]. Vohra et al. conducted a study in Canada, including a population aged 12 to 18 with resistance to prior mental health treatments, reporting a medium positive effect on stress, as evaluated using the PSS questionnaire Perceived Stress Scale) [38]. Díaz-González et al. conducted a study in Spain with adolescents aged 13 to 16 with mental health disorders, showing a medium effect on reducing anxiety levels, as evaluated using the STAI (State-Trait Anxiety Inventory) [39]. Weintraub et al. conducted a United States-based study on adolescents with depression (13 to 17 years old), suggesting medium efficacy in reducing depressive symptoms, as evaluated using the CDRS (Children’s Depression Rating Scale) [40].

The MBSR interventions varied from 8 to 13 weeks, with participant groups compared to controls receiving the usual treatment, CBT, or no intervention.

The effect sizes, measured via mean difference (MD) and confidence intervals, generally indicated moderate improvements in psychological outcomes among MBSR participants compared to controls, without statistically significant results

#### 3.1.2. MBCT

Syeda et al. conducted a study with Canadian children aged 9 to 12 with anxiety disorders, reporting a medium positive effect on anxiety symptoms, as evaluated with MASC-2 (Multidimensional Anxiety Scale for Children-2) [41]. Laundy et al. performed a Swedish study of youth aged 9 to 14 with anxiety and depressive disorders, demonstrating medium improvements in depression and strong improvements in anxiety one year post-intervention, as evaluated with the BYI (Beck Youth Inventory) [42]. Areskoug Sandberg et al. conducted a Swedish study with youth aged 9 to 16, showing moderate reductions in depression and anxiety symptoms, as evaluated with the BDI-Y (Beck Depression Inventory for Youth) and the BAI-Y (Beck Anxiety Inventory for Youth), respectively [43]. Kuyken et al. performed a study involving United Kingdom students aged 11 to 16 with mixed outcomes, suggesting no significant improvements in depression or anxiety, as evaluated with the CES-D (Center for Epidemiologic Studies Depression Scale) and the RCADS (Revised Children’s Anxiety and Depression Scale), respectively [29]. Peter et al. conducted a study with Indian adolescents aged 12 to 14 with anxiety, demonstrating a strong positive effect in reducing anxiety, as evaluated with the SCAS [44]. Volanen et al. performed a study involved Finnish students aged 12 to 15, showing small positive effects on depression when compared to controls, as evaluated with the RBDI (Revised Beck Depression Inventory-Short) [45]. Finally, Liu et al. performed a study with Chinese adolescents aged 12 to 18, demonstrating strong improvements in depression, as evaluated with the DASS-21 (Depression, Anxiety and Stress Scale-21) [46].

The MBCT interventions ranged from 4 to 28 weeks, with the control groups receiving no intervention, relaxation programs, or the usual treatment.

MBCT was effective in reducing anxiety, depression, and stress symptoms across various measures. However, there is high variability in the effect sizes (ranging from strong to negligible). Notably, there are some statistically significant results for anxiety [42,44] and depression [46].

#### 3.1.3. MBSR and MBCT

Johnson et al. [47] conducted a study in Australia with students (mean age of 13.63 ± 0.43 years). The combined intervention lasted 8 weeks, and the participants in the intervention group were compared to a control group receiving no intervention. The results demonstrated a small positive effect on depression and no significant improvements on anxiety, as evaluated with the DASS-21.

#### 3.1.4. MBIs Not Specified

Malboeuf-Hurtubise et al. [48] performed a study in Canadian students (aged 8 to 12). The mindfulness practices were developmentally appropriate to match the elementary school students’ shorter attention spans [49]. The intervention involved MBI, lasting 10 weeks, and the control group received no intervention. The intervention showed a moderate positive effect on depression and on anxiety, as evaluated with the BASC-II (Behavior Assessment System for Children-II).

### 3.2. Meta-Analysis

#### 3.2.1. Effectiveness of Mindfulness Interventions in Depression

**Figure 2:**
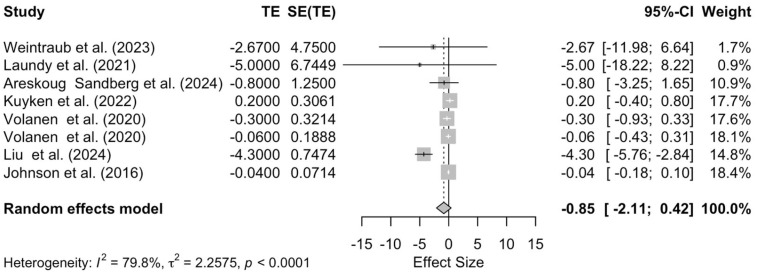
Forest plot assessing the effectiveness of mindfulness interventions on depression [29,40,42,43,45,46,47].

The results of the meta-analysis showed no significant overall effect of mindfulness interventions on depression, regardless of the specific intervention (effect size = −0.85; 95% CI −2.11 to 0.42; *p* = 0.19, Figure 2) Substantial heterogeneity was identified among the analyzed studies (τ^2^ = 2.26; I^2^ = 79.8%), which should be noted.

#### 3.2.2. Effectiveness of Mindfulness Interventions in Anxiety

**Figure 3:**
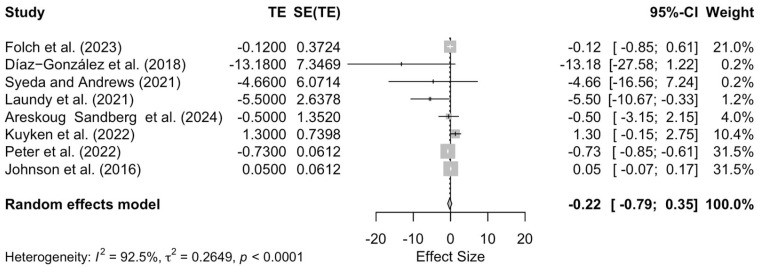
Forest plot assessing the effectiveness of mindfulness interventions on anxiety [29,37,39,41,42,43,44,47].

The results of the meta-analysis indicated no significant overall effect of mindfulness in anxiety, irrespective of the specific intervention type (effect size = −0.22; 95% CI −0.79 to 0.35; *p* = 0.45, Figure 3). Note the considerable amount of heterogeneity across the studies (τ^2^ = 0.26; I^2^ = 92.5%).

#### 3.2.3. Effects of MBCT in Depression and Anxiety

**Figure 4:**
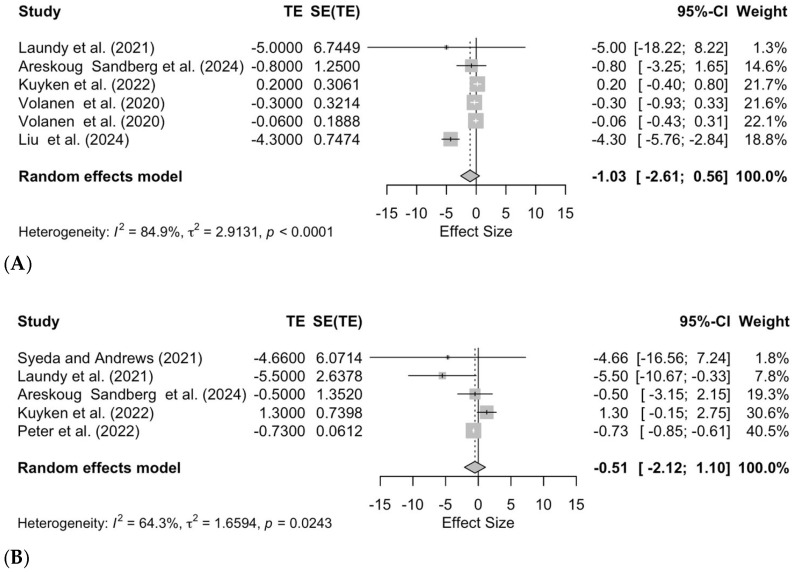
Forest plots assessing the effectiveness of MBCT on (**A**) depression [29,42,43,44,45,46] and (**B**) anxiety [29,41,42,43,44].

The subgroup analysis demonstrated that MBCT had no significant effect on depression (effect size = −1.03; 95% CI −2.61 to 0.56; *p* = 0.20, Figure 4A) or anxiety (effect size = −0.51; 95% CI −2.12 to 1.10; *p* = 0.54, Figure 4B). Note that a considerable amount of heterogeneity was observed in both subgroup analyses (depression: τ^2^ = 2.91; I^2^ = 84.9%; anxiety: τ^2^ = 1.66; I^2^ = 64.3%).

#### 3.2.4. Effects of MBSR in Depression and Anxiety

**Figure 5:**
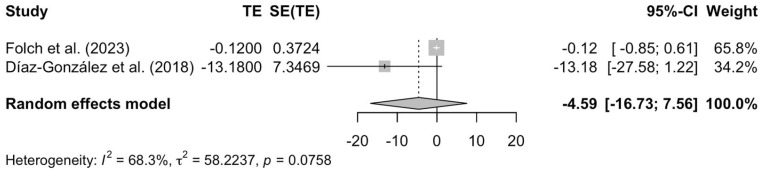
Forest plot assessing the effectiveness of MBSR on anxiety [37,39].

The subgroup analysis indicated that MBSR had no significant effect on anxiety (effect size = −4.59; 95% CI −16.73 to 7.56; *p* = 0.46, Figure 5). Once more, note that considerable heterogeneity was observed (τ^2^ = 58.22; I^2^ = 68.3%). A subgroup analysis assessing the effect of MBSR on depression was not conducted because only one study was available.

### 3.3. Risk of Bias

**Figure 6:**
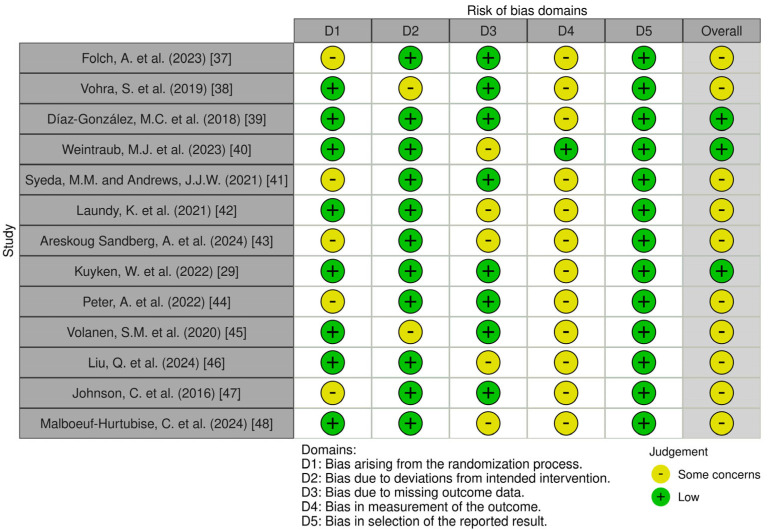
Risk of bias, represented in five categories and with its overall result, for each randomized clinical trial included in the review, ordered by intervention and age (as done in Table 1) [29,37,38,39,40,41,42,43,44,45,46,47,48].

The graphical representations of the risk of bias of the analyzed studies are shown in Figure 6. A total of 10 studies revealed some concerns and 3 studies were classified as having a low risk of bias.

## 4. Discussion

This systematic review and meta-analysis evaluated the effectiveness of Mindfulness-Based Interventions (MBIs) in improving mental health outcomes, specifically anxiety, depression, and stress, in children and adolescents. While some individual studies reported moderate to strong effects of Mindfulness-Based Stress Reduction (MBSR) and Mindfulness-Based Cognitive Therapy (MBCT) on these outcomes, the meta-analysis did not find significant overall effects on anxiety or depression. These findings highlight the variability in mindfulness intervention efficacy across different populations and the huge variety of study designs, namely in the neuropsychological tools used to evaluate the effects, suggesting that key moderating factors, such as the intervention type, duration, participant age, and baseline symptom severity, may influence results [27,28,29]. It is important to recognize that these findings may not apply universally to all children and adolescents with mood disorders. Instead, each mindfulness technique should be individualized to the specific context and precisely tailored to the unique needs of the participants.

Mindfulness interventions have been widely studied in adult populations, showing significant benefits in stress reduction, emotional regulation, and resilience [50,51,52]. However, the results in children and adolescents appear to be more variable. Some studies indicate that MBIs can foster emotional stability, enhance cognitive performance, and improve self-regulation, all of which are critical for academic success and social functioning [28,53,54]. Nevertheless, the effectiveness of these interventions is contingent on numerous factors, including the specific mindfulness approach used, the level of engagement from participants, and the expertise of facilitators delivering the programs [25,26,29], highlighting the complexity of applying these practices across different developmental stages. Furthermore, health systems face important challenges that condition the application of MBIs in youth services, particularly regarding the regional inequalities, the scarcity of human resources, and the need to increasingly and dynamically adapt to the new needs of children and young people [55]. In fact, the service organization plays an important role in optimizing patient outcomes and without equal improvements at the structural level, even promising pharmacologic advances can fall short [55].

While our meta-analysis did not show overall significant effects of MBIs on anxiety or depression—with wide variation across studies—several individual studies reported positive outcomes. For instance, even though subgroup analyses indicated that MBCT did not significantly improve depression or anxiety, some studies highlighted meaningful benefits in certain contexts. In this context, Liu et al. [46] reported a strong improvement in depression using the DASS-21 scale, while Peter et al. [44] found a significant reduction in anxiety using the SCAS scale. Conversely, Kuyken et al. [29] found no substantial benefits of school-based MBCT interventions compared to the usual care, indicating that MBCT may be less effective in universal prevention settings. Similarly, MBSR showed no significant impact on anxiety, and due to limited studies, its effect on depression could not be assessed. However, individual studies, such as those by Folch et al. [37] and Vohra et al. [38], demonstrated moderate improvements in anxiety and stress, respectively, suggesting that certain populations may benefit more from MBSR than others.

The observed heterogeneity may stem from several factors: the studies differed in terms of intervention length, session frequency, facilitator expertise, and whether they were delivered in school-based or clinical settings [5,25]. Furthermore, some studies compared MBIs to waitlist controls, while others used active interventions, such as Cognitive Behavioral Therapy (CBT) or relaxation techniques, which may dilute the observed effects [26,28,29]. Age, baseline symptom severity, and individual differences in mindfulness receptivity likely influenced outcomes, with studies with clinical populations (e.g., those diagnosed with anxiety or depression) tending to show greater improvements than those with non-clinical samples [5,27,28]. Very diverse tools were used to assess anxiety and depression in the different studies, which could have introduced measurement inconsistencies [30,45]. Additionally, the cultural context in which mindfulness interventions are implemented may play a role in determining their efficacy, with some studies suggesting that mindfulness techniques may be more accepted and effective in societies with existing traditions of meditation and self-awareness practices [25,56]. In support, the two studies with oriental populations (India and China) presented with a higher efficacy of the interventions. Studies that reported greater adherence to mindfulness practices tended to show more positive results, indicating that self-practice outside of structured sessions may be a key factor in success [27,56].

Despite its numerous benefits, mindfulness practice can occasionally lead to unintended effects, such as increased false-memory recall, temporary pain exacerbation, or heightened anxiety and agitation, particularly in individuals with an increased awareness of bodily sensations [57]. Consequently, it is essential to consider factors, such as individual characteristics, personal preferences, and medical history, when implementing these programs. Thus, a multidisciplinary approach may be necessary to implement mindfulness interventions in vulnerable populations, such as children and adolescents, to ensure a personalized approach that addresses everyone’s unique needs while integrating complementary therapeutic strategies and support systems.

Given the promising yet inconsistent findings, future research should focus on the following: standardizing MBI protocols for each specific situation to ensure consistency across studies, conducting long-term follow-ups to assess whether the benefits of MBIs are sustained over time [58,59], identifying which subgroups (e.g., clinical vs. non-clinical populations) benefit most from specific mindfulness interventions [5,27,28], comparing MBIs to other evidence-based psychological interventions, such as CBT, to determine relative efficacy [29,30], and examining the role of digital mindfulness interventions, as app-based programs and virtual mindfulness training are increasingly popular among youth and may provide scalable and accessible solutions for mental health support [60,61]. Furthermore, it would be interesting to perform a questionnaire about perceived satisfaction with this type of intervention for children and adolescents, involving specific clinical interviews to understand the symptomatology of children and adolescents knowing that they could be subjected to the bias of social desirability, and for their parents, since parents should be involved as proxy reporters of their children’s and adolescents’ anxiety and depression.

The limitations of the present systematic review and meta-analysis include the following: significant heterogeneity in the study designs, intervention protocols, and outcome measures; limited sample sizes; the potential publication bias, with some concerns for most included studies that may affect the applicability of the conclusions; the possibility that null or negative studies are underrepresented in the literature; and the inconsistent reporting of participant characteristics. Despite the lack of significant overall effects, individual studies suggest that MBIs, particularly MBCT, may offer valuable tools for youth mental health support, particularly when tailored to specific needs.

## 5. Conclusions

This systematic review and meta-analysis provide a comprehensive and up-to-date evaluation of the positive impact of MBIs on anxiety and depression in children and adolescents. While individual studies report promising effects, this meta-analysis did not find statistically significant overall benefits. The high heterogeneity observed underscores the need for further research to identify patient-level moderators and contextual factors that influence response to MBIs in youth.

Overall, while MBIs may not be a universal solution, they represent a valuable addition to the array of mental health interventions available for children and adolescents. Continued refinement and rigorous evaluation of mindfulness programs will be essential in maximizing their potential benefits. MBIs should be considered a promising complementary rather than primary treatment approach, pending further evidence.

## Figures and Tables

**Table 1:** Comprehensive overview of the key characteristics of the included studies, ordered by intervention and age.

Authors (Year)	Country	Sample Characteristics	Total N	Age (Years)	Intervention (N)	Control (N)	Period of Intervention	Outcome Measure	Effect Size (95% CI)	Effectiveness of Mindfulness Interventions
Folch, A. et al. (2023) [37]	Spain	Female and male students in 5th or 6th grade	100	9 to 11	MBSR (69)	No intervention (31)	13 weeks	SPECI	MD = −0.12 (−0.85, 0.61)	+
Vohra, S. et al. (2019) [38]	Canada	Female and male students with a history of not responding to previous mental health interventions and requiring a high degree of intensive treatment	81	12 to 18	MBSR (42)	Usual treatment (39)	10 weeks	PSS	MD = −0.88 (−4.08, 2.32)	+
Díaz-González, M.C. et al. (2018) [39]	Spain	Female and male students with mental health disorders	80	13 to 16	MBSR (41)	Usual treatment (39)	8 weeks	STAI	MD = −13.18 (−27.58, 1.22)	+
Weintraub, M.J. et al. (2023) [40]	United States of America	Female and male students with a mood disorder, namely depression	54	13 to 17	MBSR (30)	CBT (24)	2 months	CDRS	MD = −2.67 (−12.01, 6.61)	+
Syeda, M.M. and Andrews, J.J.W. (2021) [41]	Canada	Female and male students with anxiety disorder	19	9 to 12	MBCT (11)	No intervention (8)	4 weeks	MASC-2	MD = −4.66 (−22.54, 1.26)	+
Laundy, K. et al. (2021) [42]	Sweden	Female and male students with mental health disorders, namely anxiety disorders and depressive disorders	34	9 to 14	MBCT (22)	No intervention (12)	8 weeks	BYI	MD = −5.00 (−18.22, 8.22), depression at 1 year post-intervention	+
									MD = −5.50 (−10.67, −0.33), anxiety at 1 year post-intervention)	+ +
Areskoug Sandberg, A. et al. (2024) [43]	Sweden	Female and male students in 3rd to 9th grade	1394	9 to 16	MBCT (902)	No intervention (492)	10 weeks	BDI-Y, depression	MD = −0.80 (−1.70, 3.20)	+
								BAI-Y, anxiety	MD = −0.50 (−2.10, 3.20)	+
Kuyken, W. et al. (2022) [29]	United Kingdom	Female and male students in 7th to 10th grade	7561	11 to 16	MBCT especially directed at students (3768)	Usual treatment (3793)	28 weeks	CES-D, depression	MD = 0.20 (−0.40, 0.80)	−
								RCADS, anxiety	MD = 1.30 (−0.20, 2.70)	−
Peter, A. et al. (2022) [44]	India	Female and male students with anxiety	65	12 to 14	MBCT (33)	No intervention (32)	12 weeks	SCAS	MD = −0.73 (−0.61, −0.85)	+ +
Volanen, S.M. et al. (2020) [45]	Finland	Female and male students in 6th to 8th grade	2254	12 to 15	MBCT (1020)	No intervention (277)	9 weeks	RBDI	MD = −0.30 (−0.93, 0.33)	+
						Relaxation program (957)			MD = −0.06 (−0.43, 0.31)	+
Liu, Q. et al. (2024) [46]	China	Female and male students in 7th to 12th grade	94	12 to 18	MBCT (45)	No intervention (49)	8 weeks	DASS-21	MD = −4.30 (−5.76, −2.83)	+ +
Johnson, C. et al. (2016) [47]	Australia	Female and male students	269	13.63 ± 0.43	MBCT/MBSR (115)	No intervention (154)	8 weeks	DASS-21	MD = −0.04 (−0.18, 0.10), depression	+
									MD = 0.05 (−0.07, 0.17), anxiety	−
Malboeuf-Hurtubise, C. et al. (2024) [48]	Canada	Female and male students in 3rd to 6th grade	231	8 to 12	MBIs, not specified (127)	No intervention (104)	10 weeks	BASC-II	Coef = 0.45 (−0.36, 1.26), depression	+
									Coef = 0.16 (−0.32, 0.64), anxiety	+

**Legend: BASC-II**, Behavior Assessment System for Children-II; **BAI-Y**, Beck Anxiety Inventory for Youth; **BDI-Y**, Beck Depression Inventory for Youth; **BYI**, Beck Youth Inventory; **CBT**, Cognitive Behavioral Therapy; **CDRS**, Children’s Depression Rating Scale; **CES-D**, Center for Epidemiologic Studies Depression Scale; **CI**, Confidence Interval; **Coef**, Coefficient; **DASS-21**, Depression, Anxiety and Stress Scale-21; **MASC-2**, Multidimensional Anxiety Scale for Children-2; **MBSR**, Mindfulness-Based Stress Reduction; **MBCT**, Mindfulness-Based Cognitive Therapy; **MD**, Mean Difference; **MBIs**, Mindfulness-Based Interventions; **N**, Number; **PSS**, Perceived Stress Scale; **RCADS**, Revised Children’s Anxiety and Depression Scale; **RBDI**, Revised Beck Depression Inventory-Short; **SCAS**, spence children’s Anxiety Scale; **SPECI**, Screening for Children’s Emotional and Behavioral Problems; **STAI**, State-Trait Anxiety Inventory; **−**, no significant improvements; **+**, medium effect; **+ +**, positive effect.

## Data Availability

All data generated during this study are available upon reasonable request from the corresponding author.

## Abbreviations

The following abbreviations are used in this manuscript:

MBIs	Mindfulness-Based Interventions
RCTs	Randomized Clinical Trials
PROSPERO	International Prospective Register of Systematic Reviews
PRISMA	Preferred Reporting Items for Systematic Reviews and Meta-Analyses
MBSR	Mindfulness-Based Stress Reduction
MBCT	Mindfulness-Based Cognitive Therapy
MSC	Mindfulness Self-Compassion
MBRP	Mindfulness-Based Relapse Prevention
MORE	Mindfulness-Oriented Recovery Enhancement
CBT	Cognitive Behavioral Therapy
CI	Confidence Interval
MD	Mean Difference
BASC-II	Behavior Assessment System for Children-II
BAI-Y	Beck Anxiety Inventory for Youth
BDI-Y	Beck Depression Inventory for Youth
BYI	Beck Youth Inventory
CES-D	Center for Epidemiologic Studies Depression Scale
DASS-21	Depression, Anxiety and Stress Scale-21
MASC-2	Multidimensional Anxiety Scale for Children-2
PSS	Perceived Stress Scale
RCADS	Revised Children’s Anxiety and Depression Scale
RBDI	Revised Beck Depression Inventory—Short
SCAS	Spence Children’s Anxiety Scale
SPECI	Screening for Children’s Emotional and Behavioral Problems
STAI	State-Trait Anxiety Inventory
